# A reinforcement learning algorithm to optimize resource utilization in combat casualty care

**DOI:** 10.1038/s41598-025-28021-6

**Published:** 2025-12-24

**Authors:** Manivannan Subramaniyan, Xin Jin, Sridevi Nagaraja, Anders Wallqvist, Jaques Reifman

**Affiliations:** 1 Department of Defense Biotechnology High Performance Computing Software Applications Institute, Defense Health Agency Research & Development, Medical Research and Development Command, ATTN: FCMR-TT, 504 Scott Street, Fort Detrick, MD 21702-5012 USA; 2 The Henry M. Jackson Foundation for the Advancement of Military Medicine, Inc., Bethesda, MD USA

**Keywords:** Artificial intelligence, Reinforcement learning, Fluid resuscitation, Hemorrhage, Resource utilization, Trauma, Computational biology and bioinformatics, Engineering, Health care, Mathematics and computing, Medical research

## Abstract

Fluid resuscitation immediately following a hemorrhagic injury improves clinical outcome. However, future military conflicts are expected to result in mass-casualty incidents with limited availability of fluid-resuscitation resources. Here, we developed and assessed the performance of a reinforcement learning AI method that optimized clinical outcome and fluid allocation under constrained resources. We generated a large cohort of synthetic trauma casualties using a validated cardio-respiratory computational model, simulating vital-sign time-series data for realistic battlefield scenarios involving hemorrhage, tourniquet application, and fluid resuscitation. For each casualty, we simulated three intervention options—infusion or no infusion of one fluid unit every 30 min over 90 min—and assessed whether the interventions restored the vital signs to “healthy” levels. Using these data, we trained the reinforcement learning model to predict the optimal sequence of interventions that maximized the number of casualties restored while minimizing fluid utilization. Using independent simulated data, we found that the AI model was twice as efficient and restored more than twice as many casualties as the current standard of care across varying numbers of casualties and resource limitations. These results highlight the model’s potential to enable personalized interventions, enhance treatment efficiency, and support automated medical decision-making in resource-constrained environments.

**Supplementary Information:**

The online version contains supplementary material available at 10.1038/s41598-025-28021-6.

## Introduction

Future armed conflicts between near-peer adversaries are expected to involve large-scale operations across multiple domains and lead to mass casualties^1^. In this scenario, combat casualty-care management systems will likely be overwhelmed by shortages of medical personnel, extended treatment times with limited evacuation opportunities, compromised communications, and limited medical supplies. Treating mass casualties in such a combat environment poses significant challenges, as casualty condition and resource availability can evolve rapidly and unpredictably. In these situations, automated systems powered by artificial intelligence (AI) technologies could aid medical personnel across the continuum of casualty care by facilitating triage, diagnosis, treatment, and resource utilization. In particular, optimal allocation of resuscitation fluids, by determining how best to prioritize transfusion of limited blood supplies, could have a large impact on casualty survival, as uncontrolled bleeding remains the leading cause of death on the battlefield^2–4^ and blood transfusion within 30 min of injury leads to better outcomes^5^.

To identify combat casualties who require resuscitation fluids, U.S. military caregivers follow the standard of care delineated by the Vampire program guidelines^6^ and the Tactical Combat Casualty Care (TCCC) recommendations^7^. The Vampire guidelines recommend administering fluid resuscitation when heart rate (HR) exceeds 100 beats/min (bpm) or systolic blood pressure (SBP) falls below 100 mmHg^6^. While robust and field-tested, these recommendations are based on the current physiological state of a casualty and do not account for mass-casualty incidents, where caregivers may need to quickly decide how best to allocate resuscitation fluids when the number of casualties exceeds the available resources. To overcome these limitations, we recently developed an AI algorithm based on recurrent artificial neural networks (RNNs) that prognosticates the physiological state of each casualty under various treatment options and determines how to optimize the clinical outcome for the largest number of casualties with limited resuscitation fluids^8^. Through simulations, we showed that this approach led to a 46% increase in the number of casualties restored to a “healthy” physiological state compared to the Vampire program. However, while this approach is viable for short treatment horizons, it becomes increasingly complex over long treatment periods, as the number of possible treatment combinations requiring prognostication grows exponentially with time. In addition, RNNs depend on a time series of vital-sign data from each casualty, which can be challenging to collect in field environments and could be laden with motion artifacts^9^. Ideally, a robust algorithm would be able to prognosticate the physiological state of a casualty based on a single vital-sign measurement at the current time.

To address these limitations, we explored an alternative approach based on reinforcement learning (RL) to optimize the allocation of limited fluid resources to multiple casualties simultaneously. RL has been previously applied to provide medical decision support, especially to optimize treatment for individual patients with conditions such as sepsis^10^, diabetes^11^, and mechanical ventilation in intensive care units^12^ (see^13,14^ for comprehensive reviews). However, these studies primarily focused on the optimal treatment of individual patients without any consideration of possible constraints on medical supplies, as encountered in mass-casualty incidents. Here, we developed an RL model capable of creating personalized fluid resuscitation plans for each casualty, while simultaneously optimizing the clinical outcome of the largest number of casualties with limited fluid resources. We used a previously validated cardio-respiratory (CR) computational model^15^ to simulate the vital-sign dynamics of moderate to severe hemorrhage and fluid resuscitation for a large cohort of synthetic battlefield trauma casualties. Using these vital-sign data, we trained RL models to predict the optimal fluid resuscitation treatment for each casualty over a 90-min treatment horizon, with interventions occurring every 30 min. We then evaluated the performance of the algorithm by comparing the fluid allocation proposed by the RL model with that of the Vampire program. We hypothesized that the RL model would lead to more efficient fluid allocation, resulting in a larger number of simulated trauma casualties restored to a healthy physiological state.

## Methods

We aimed to develop a fluid-allocation algorithm that restored the vital signs of the largest possible number of hemorrhagic patients to healthy levels over a 90-min treatment horizon, while using the least amount of available fluids. To this end, we *1*) generated vital-sign trajectories of synthetic trauma casualties following hemorrhage and fluid resuscitation; *2*) used a portion of these data to train an RL model; and *3*) used the remaining data to assess our hypothesis by comparing the performance of the model against the fluid allocation recommended by the Vampire program, which is the current standard of care. As the outcome measures, we computed the fraction of casualties restored by the RL model relative to those achieved by the Vampire program and the relative fluid-utilization efficiency between the two methods.

### Step 1. Generation of a cohort of synthetic trauma casualties

#### CR model

The CR model^15^ represents the cardiovascular and respiratory systems and their regulatory mechanisms (via 74 ordinary differential and algebraic equations with 74 parameters) and has been previously demonstrated to provide adequate predictions of vital signs for simulated battlefield trauma casualties^15^. The inputs to the model include hemorrhage rate, fluid-infusion rate, minute ventilation, and fraction of inspired oxygen, while the model outputs comprise HR, arterial blood pressure (SBP, diastolic, and mean), partial pressure of end-tidal carbon dioxide, and oxygen saturation. The vital-sign data used in this study only included HR and SBP.

#### Creation of hemorrhage and treatment scenarios

**Fig. 1:**
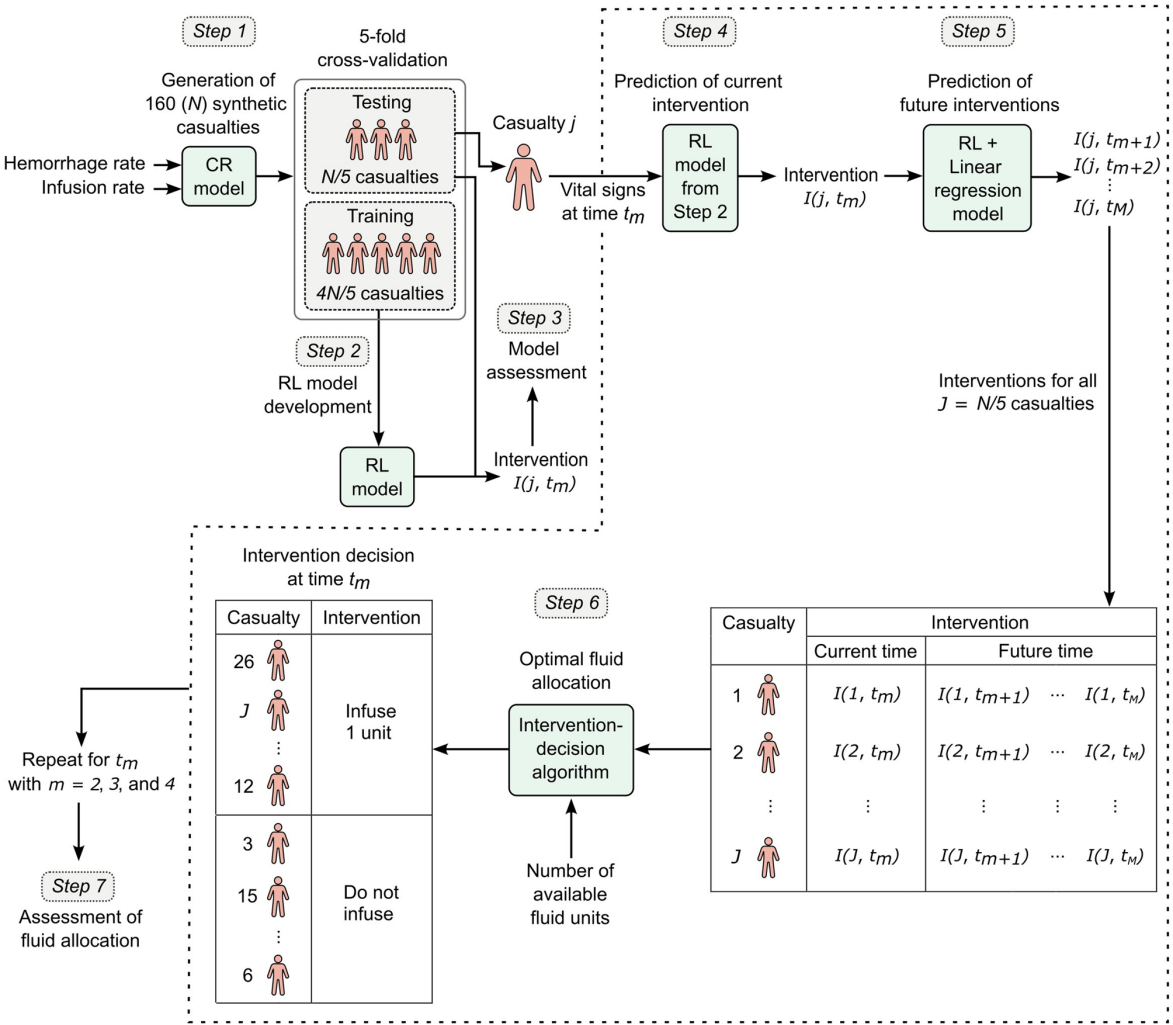
Overview of the reinforcement learning (RL) model to optimize fluid allocation for a cohort of synthetic trauma casualties. The optimization involved the following steps. *Step 1*: We generated 160 (*N*) synthetic trauma casualties using a cardio-respiratory (CR) computational model, which takes hemorrhage rate and fluid infusion rate as inputs and produces vital signs as outputs. To perform 5-fold cross-validation, we created five distinct data folds, each fold consisting of a random partitioning of the data into training (4*N*/5 casualties) and testing (*N*/5 casualties) datasets. *Step 2*: Using the training datasets, we developed an RL model capable of predicting the optimal fluid-infusion intervention *I*(*j*, *t*_*m*_) for casualty *j* at intervention time *t*_*m*_, with *m* = 2, 3, …, *M*, where *M* denotes the final intervention time in the treatment horizon. *Step 3*: Using the testing dataset, for each casualty, we assessed the accuracy of the RL model to predict the sequence of actions requiring the least amount of fluids, as determined by the CR model. *Steps 4 − 6*: At intervention time *t*_*m*_, we allocated available fluids optimally to the cohort of *J* (*N*/5) casualties in the testing dataset. Specifically, for each casualty *j*, the RL model predicted the optimal intervention at the current time *t*_*m*_ (*Step 4*) based on current vital signs, and in combination with a linear regression model, it predicted the optimal interventions for future time points *t*_*m+*1_, *t*_*m+*2_, …, *t*_*M*_ (*Step 5*). Using both current and projected interventions along with fluid availability, we prioritized casualties and determined personalized intervention decisions (i.e., infuse 1 unit of fluid or do not infuse) at time *t*_*m*_ via an intervention-decision algorithm (*Step 6*). We repeated *Steps* 4–6 at subsequent time points *t*_*m+*1_, *t*_*m+*2_, …, *t*_*M*_. *Step 7*: We assessed the ability of the fluid-allocation algorithm to restore the vital signs of the hemorrhagic casualties to healthy levels at the end of the treatment scenario. Then, we repeated *Steps* 2–7 for each of the remaining four folds created in *Step 1*, averaging the results across all five folds.

By using different combinations of hemorrhage and CR-model parameter settings, each representing a different trauma “casualty,” we created synthetic vital-sign data for a large cohort of trauma casualties with moderate (Class II) to severe (Class III) hemorrhage^16^, followed by variable durations of tourniquet application and fluid-infusion treatment (Fig. 1, *Step 1*) in accordance with the guidelines for pre-hospital treatment of combat casualties^6^. With respect to the CR model, the terms “bleeding” and “hemorrhage” correspond to fluid being lost at a constant rate for a defined period of time, and applying a “tourniquet” refers to setting the bleeding rate to zero. This simplified hemorrhage case parallels those of compressible extremity bleeding, which can be controlled by tourniquet application.

**Fig. 2:**
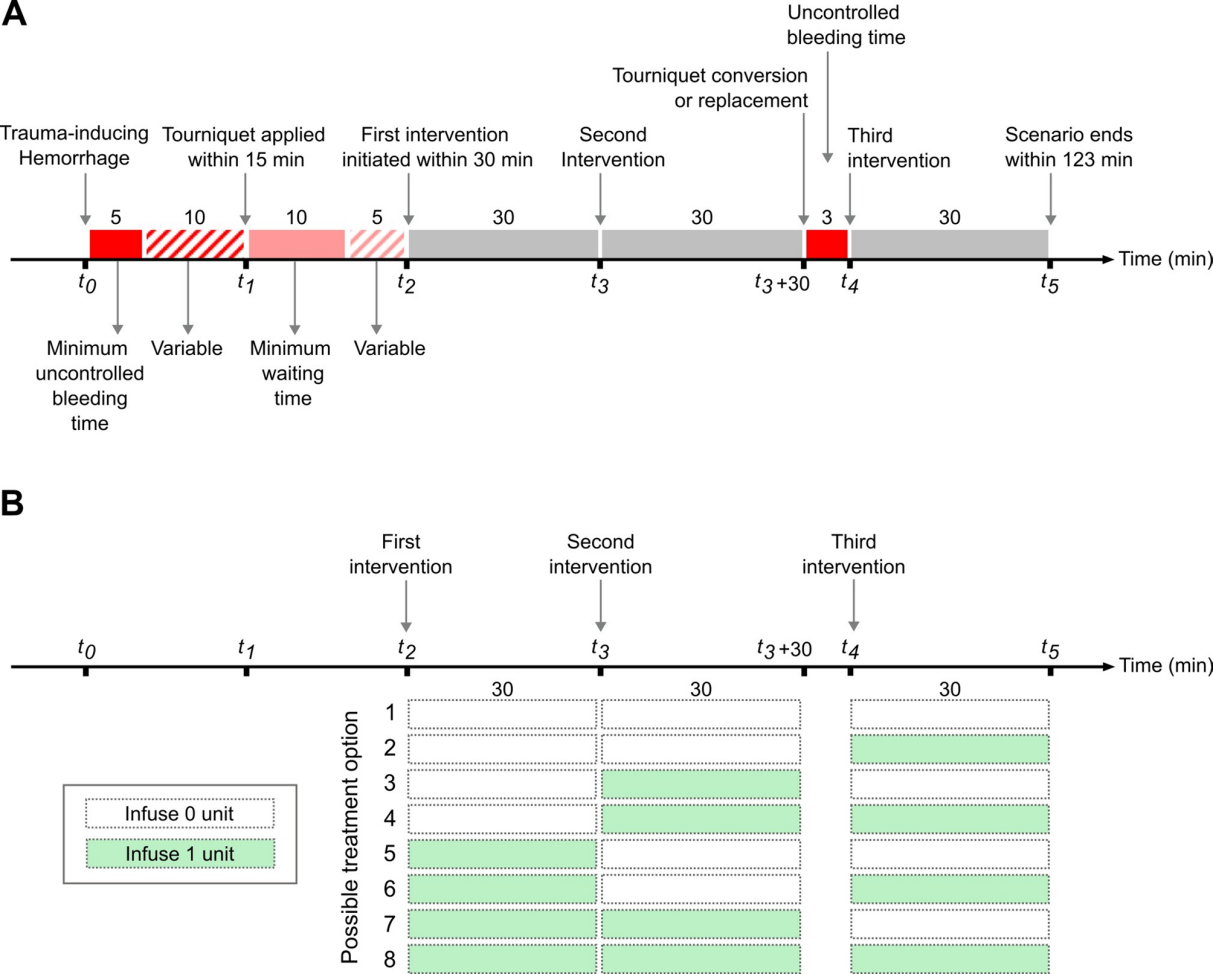
Simulated study protocol to assess the performance of the reinforcement learning model. **A**) Timeline of events and actions for hemorrhage and fluid treatment interventions and **B**) the eight possible fluid-infusion interventions. Numbers immediately above boxes indicate time intervals in minutes.

Figure 2A shows the timeline of the following sequence of hemorrhage and treatment scenarios simulated in this study: *1*) fixed 5-min uncontrolled bleeding initiated at time *t*_*0*_ to simulate trauma-induced hemorrhage; *2*) variable 0- to 10-min period of additional bleeding followed by tourniquet application at time *t*_*1*_ to stop the compressible bleeding; *3*) variable 10- to 15-min period, with the first fluid-infusion intervention initiated at time *t*_*2*_ (within 30 min of the traumatic injury), followed by a second 30-min fluid-infusion intervention initiated at time *t*_*3*_; *4*) 3 min of hemorrhage to simulate temporary bleeding during tourniquet replacement or conversion, followed by tourniquet application at the end of the 3 min to stop the compressible bleeding; and *5*) 30-min fluid-infusion intervention, starting at time *t*_*4*_ and completion of the scenario at time *t*_*5*_. See the **Supplementary Information** for the guidelines used for selecting the above time periods.

We set the bleeding volume in the range of 0.75–2.00 L to limit the bleeding rate to the highest reported rate of 0.22 L/min^17–19^. Given that a maximum of two units of whole blood or blood products are generally administered over a 60-min period^6^, which is equivalent to infusion of one unit over 30 min, we considered eight different treatment options for the 90 min of treatment time (60 min from *t*_*2*_ to *t*_*3*_ + 30 plus 30 min from *t*_*4*_ to *t*_*5*_) arising from the combination of two possible treatment options—infusion of 0 or 1 unit—at each of the three 30-min infusion periods (Fig. 2B). With the volume of each unit being approximately 0.55 L, we set the infusion rate to 1.10 L/h. Finally, we assessed the performance of the AI model by evaluating the vital signs of each casualty at *t*_*5*_, which corresponded to no more than 123 min after the initial traumatic injury.

#### Generation and use of synthetic vital-sign data

We created a cohort of trauma casualties corresponding to the scenario in Fig. 2A. To create different vital-sign trajectories for different casualties with different hemorrhage levels, we generated unique parameter sets of the CR model to construct a cohort of 160 synthetic casualties (see the **Supplementary Information** for details). For each casualty, we applied each of the eight possible treatment options in Fig. 2B, resulting in a total of 8 × 160 vital-sign trajectories to train and test the RL model. To this end, we applied a 5-fold cross-validation procedure, where we randomly partitioned the 160 (*N*) casualties into five folds (32 casualties/fold). We used the data from four of the five folds to train the RL model (Fig. 1, between *Steps 1* and *2*) and the data from the fifth fold to test the model. We tested the model by assessing its accuracy in predicting the sequence of actions for each casualty that consumed the least amount of fluids over the entire scenario, as determined by the CR model (Fig. 1, *Step 3*), and its ability to restore the largest possible number of casualties to a healthy target range in vital-sign space at time *t*_*5*_, while using the least amount of fluids (Fig. 1, *Step 7*).

### Step 2. RL model development

The goal of the RL model is to determine the optimal fluid-infusion intervention decision at times *t*_*2*_, *t*_*3*_, and *t*_*4*_ in order to restore the vital signs of each trauma casualty to the “healthy target range”—as defined by the Vampire program (HR ≤ 100 bpm and SBP ≥ 100 mmHg)—by the end of the treatment scenario at time *t*_*5*_, while using the least amount of fluids. To achieve this goal, we formulated the RL problem as a Markov decision process, where an agent observes the current casualty state “*s*_*t*_” at time $\it \mathrm{t}$ and performs an action “$\it \mathrm{a}$,” which results in a transition to the next state “*s*_*t+1*_” and a reward “$\it \mathrm{r}$,” where $\it \mathrm{r}\in\:\mathbb{R}$. An agent is any entity that learns how to make optimal actions at each state *s*_*t*_ so as to maximize the expected return or discounted future reward $\it \mathrm{r}\:$^20^. We defined three states *s*_*t2*_, *s*_*t3*_, and *s*_*t4*_ corresponding to intervention times *t*_*2*_, *t*_*3*_, and *t*_*4*_, respectively, in which the agent took actions that resulted in a terminal state *s*_*t5*_ at time *t*_*5*_, where no action was taken. We defined the state *s*_*t*_ at a given time *t*_*m*_ by the casualty’s vital signs HR and SBP at *t*_*m*_ as well as the vital signs at the previous intervention time *t*_*m−1*_ and the number of fluid units used in each of the past two interventions. In a real-world setting, the state at a given time would represent the physiological status of a casualty with potentially different patterns of compressible hemorrhage controlled by the application of a tourniquet. We defined two actions: $\it \mathrm{a}$ = 0 and $\it \mathrm{a}$ = 1, where 0 denotes no fluid infusion for the next 30 min and 1 represents infusion of 1 unit for the next 30 min. The eight treatment options in Fig. 2B correspond to the eight possible sequences of actions the agent could take during the entire treatment scenario. Therefore, to obtain vital-sign values for states resulting from the agent’s actions, we used the CR model to generate vital signs for the corresponding treatment option. To encourage the agent to use the least amount of fluids required to restore a casualty, we defined a reward function $\it \mathrm{r}$ that favored actions that improved the resulting vital signs and discouraged actions that increased fluid usage (**Supplementary Information**, Eq. 1).

To start the training of the RL model, for each of the training dataset casualties, the agent started at state *s*_*t2*_, took a sequence of actions, and completed a learning *episode* after reaching the terminal state *s*_*t5*_. Choosing the best action at each state so as to maximize the discounted future reward involved learning the optimal action-value function $\:{\mathrm{Q}}^{*}($*s*_*t*_,$\textit{a})$, which predicted the value of taking action $\it \mathrm{a}$ in state *s*_*t*_. We then sought to estimate $\:{\mathrm{Q}}^{*}($*s*_*t*_,$\textit{a})\:$using a feedforward artificial neural network (Q-network) consisting of two hidden layers with 64 and 32 units each. This network used leaky-ReLU activations, with batch normalization applied to the outputs of each layer^21^. The input to the network was the state *s*_*t*_ and the outputs were the $\:{\mathrm{Q}}^{*}($*s*_*t*_,$\textit{a})$ values for actions $\it \mathrm{a}$ = 0 and $\it \mathrm{a}$ = 1. We trained this network using the information from the learning episodes, as previously described^22,23^. See the **Supplementary Information** for a detailed description of the RL model development.

### Step 3. RL model assessment.

After training the RL model, we assessed the predicted fluid allocations by computing the fraction of casualties in the testing dataset for whom the predicted sequence of actions matched the “theoretical optimum.” As the theoretical optimum, we selected the sequence among the eight possible treatment options in Fig. 2B that consumed the least amount of fluids to restore a casualty’s vital signs to the healthy target range, as determined by the CR model. See the **Supplementary Information** for additional details.

### Step 4. Prediction of the current intervention

To determine the optimal action at each intervention time point *t*_*2*_, *t*_*3*_, and *t*_*4*_, we needed to estimate the total number of fluid units (one, two, or three) required by each casualty over the entire treatment period. Therefore, we first computed the optimal action at the current intervention time point *t*_*m*_ (*Step 4*) and then at future intervention time points *t*_*m+1*_, with $\it \mathrm{m}$ = 2 and 3 (*Step 5*).

For each casualty *j* in the testing dataset, we first determined the optimal action (i.e., intervention) $$\textit{I}(j,t_m)$$ at the current intervention time *t*_*m*_ (Fig. 1, *Step 4*). Specifically, we computed $$\:{\mathrm{Q}}^{*}($$*s*_*t*_,$$\textit{a})$$ values for the two possible actions (0 and 1) by providing the state *s*_*t*_ at the current time *t*_*m*_ as input to the Q-network and selected the action (0 or 1) that resulted in the higher value as the intervention $$\textit{I}(j$$, *t*_*m*_$$)$$.

### Step 5. Prediction of future interventions

In a real-world application, we would only have access to vital-sign values at the current time $$\it {\mathrm{t}}_{\mathrm{m}}$$ to compute $$\textit{I}(j$$, *t*_*m*_$$)$$. To estimate interventions $$\textit{I}(j$$, *t*_*m+1*_$$)$$, …, $$\textit{I}(j$$, *t*_*M*_$$)$$ at future time points would require knowledge of future values of vital signs to determine their states. Hence, to predict future vital signs and states for *t* > *t*_*m*_ so that we can use the Q-network to determine optimal actions for future interventions, we trained a linear regression model to predict the next state *s*_*t+1*_ given the current state *s*_*t*_ and action $$\it \mathrm{a}$$. Given a state-action pair at *t*_*m*_, we recursively applied the linear regression model to predict the next state, and then used the Q-network to compute the optimal action for the predicted state. By repeating this process, we determined the sequence of optimal interventions $$\textit{I}(j$$, *t*_*m+1*_$$)$$, …, $$\textit{I}(j$$, *t*_*M*_$$)$$ for all intervention time points up to *t*_*M*_, for each of the $$\it \mathrm{N}$$/5 casualties in the testing dataset (Fig. 1, *Step 5*).

### Step 6. Optimal fluid allocation

In a resource-constrained environment, to maximize the number of casualties whose vital signs could be restored to the healthy target range after the three 30-min interventions, an optimal allocation strategy should avoid the administration of fluids to casualties whose vital signs cannot be restored even with three units and prioritize fluid infusion to casualties requiring one unit, followed by those needing two units, and then those requiring all three units. Applying this strategy, we proceeded to allocate available fluids to casualties based on the following intervention-decision algorithm (Fig. 1, *Step 6*): *1*) for *intervention decisions at t*_*2*_, we used the predicted interventions $$\textit{I}(j$$, *t*_*2*_$$)$$, $$\textit{I}(j$$, *t*_*3*_$$)$$, and $$\textit{I}(j$$, *t*_*4*_$$)$$ for each casualty $${j}$$ in *Step 5* to sort the casualties in ascending order of the total number of fluid units required in the three interventions. Then, we allocated fluids to those needing one unit. Note that a casualty may not need that one unit at the current time *t*_*2*_ but may need it at a later intervention time point (*t*_*3*_ or *t*_*4*_). In this case, we “reserved” one unit of fluid for future use. Next, depending on the amount of fluid remaining, we allocated fluid to as many casualties needing two units as possible, while reserving a second unit of fluid for each casualty for future use. Finally, if fluids were still available, we allocated them to casualties needing three units, while reserving two additional units for each casualty for future use. *2*) For *intervention decisions at t*_*3*_, we repeated the above strategy by computing the total number of fluid units needed for restoring vital signs and prioritizing allocation of fluids (including those reserved at time *t*_*2*_) to casualties needing one unit and then to those needing two units. *3*) For *intervention decisions at t*_*4*_, we allocated the remaining fluids to casualties who needed one unit.

### Step 7. Assessment of fluid allocation

Using the testing dataset, we assessed the ability of the RL model to optimize fluid allocation under resource-limited conditions by comparing its performance against that of the Vampire program. The Vampire program is the U.S. Department of Defense standard of care for blood transfusion after a traumatic event^6^ based on the values of two vital signs (HR and SBP) and the presence of amputation. We used the CR model to predict the resulting vital signs after each fluid-infusion intervention proposed by the Vampire program. However, because the CR model does not explicitly consider amputations, we focused our analysis on HR and SBP and modified the Vampire program into a three-step process^8^: *1*) at time *t*_*2*_, if the vital signs of the casualty were not within the healthy target range, we initiated infusion of one unit of fluid over 30 min; *2*) at time *t*_*3*_, we infused an additional unit of fluid if the vital signs, as predicted by the CR model, did not fall within the healthy target range; and *3*) at time *t*_*4*_, we infused a third unit of fluid if the vital signs did not fall within the healthy target range after the administration of the second fluid unit.

To obtain an upper bound on the maximum number of possible restored casualties, we used the CR model to determine the optimal fluid treatment for each casualty and prioritized allocation to casualties requiring one unit, followed by those requiring two units, and finally those requiring three units. Then, we performed two types of analyses.

#### Analysis 1

For a fixed number of 32 casualties, we assessed the subset of casualties restored at time *t*_*5*_ as a function of varying units of available fluid. We compared the RL model versus the Vampire program as well as the best-possible results obtained with the CR model. Next, we compared the number of units of fluid used by the RL model and the Vampire program in excess of the fewest-possible allocation required by the CR model to restore the casualties.

#### Analysis 2

We examined how the RL and Vampire methods performed when we allocated varying units of available fluid to varying numbers of casualties. To achieve this, we partitioned the 32 casualties into two groups of 16 casualties, four groups of eight casualties, and eight groups of four casualties, and used both methods to allocate fluid to each group and averaged the results across the groups for each partition. Then, we computed *1*) the fraction of casualties restored by the RL model compared to the Vampire program and *2*) the relative ratio $\textit{R}$ of fluid-utilization efficiency between the two methods, as defined by $\textit{R}\:\mathrm{=}\:\mathrm{[(}{\textit{N}}_{\textit{A}}\mathrm{/}{\textit{U}}_{\textit{A}}\mathrm{)/(}{\textit{N}}_{\textit{V}}\mathrm{/}{\textit{U}}_{\textit{V}}\mathrm{)]}$, where ${\textit{N}}_{\textit{A}}$ and ${\textit{N}}_{\textit{V}}$ denote the total number of casualties restored by the RL and Vampire allocations, respectively, and ${\textit{U}}_{\textit{A}}$ and ${\textit{U}}_{\textit{V}}$ represent the total number of available units of fluid utilized by each method. Hence, $\textit{R}$ >1.00 indicates a greater allocation efficiency of the RL model over the Vampire program.

### Sensitivity analysis

To assess the sensitivity of the fluid-allocation decisions to small errors in vital-sign measurements, we added normally distributed noise with zero mean and standard deviation of 3.0 to the vital signs generated by the CR model and repeated the analyses. The selected noise level reflects the accuracy of representative Food and Drug Administration-cleared vital-sign monitors (± 3.0 bpm for HR and ± 3.0 mmHg for blood pressure)^24^.

## Results

### Characteristics of the synthetic casualties

**Fig. 3:**
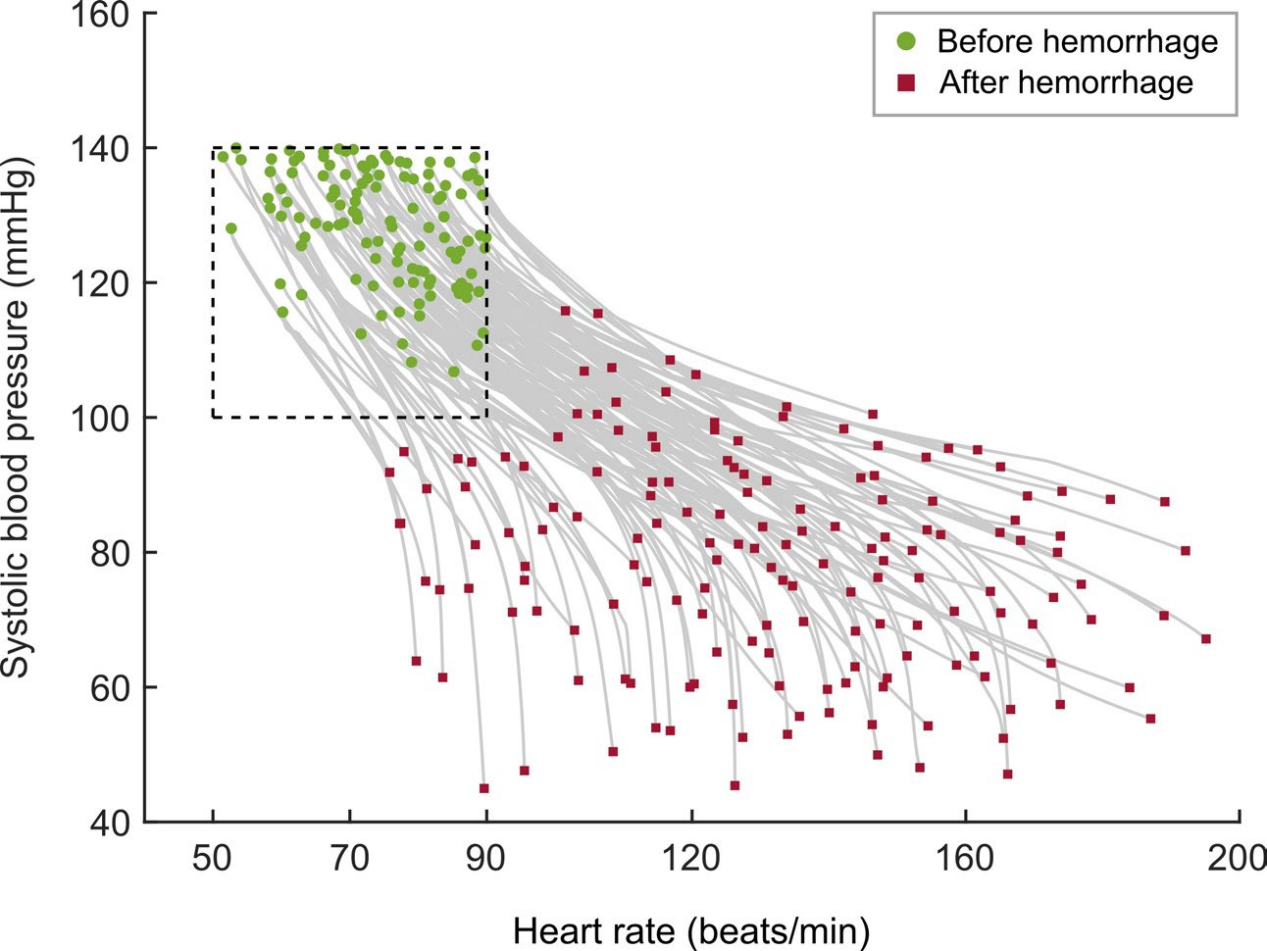
Simulated vital-sign trajectories of 160 synthetic casualties following trauma-induced hemorrhage, before the application of a tourniquet. The beginning (filled circles) and end (filled rectangles) of the trajectories correspond to times *t*_*0*_ and *t*_*1*_, respectively, in Fig. 2A. The range within the dashed lines represents the initial healthy vital-sign range.

For the 160 synthetic trauma casualties used in the study, Fig. 3 shows the distribution of vital-sign trajectories corresponding to the uncontrolled bleeding period between *t*_*0*_ and *t*_*1*_. The set of initial vital signs covered a large portion of the healthy initial range (50 ≤ HR ≤ 90^25^ and 100 ≤ SBP ≤ 140^6,26^), although the region corresponding to HR ≤ 70 and SBP ≤ 120 was sparsely populated because a large number of casualties in this region exhibited non-physiological vital signs under severe bleeding, which led to their exclusion during the down-selection process.

The application of hemorrhage scenarios, as expected, resulted in an increase in HR and a decrease in SBP at time *t*_*1*_, with HR ranging from 70 to 200 bpm and SBP ranging from 40 to 120 mmHg, which covered the full span of their respective physiological limits. Based on the CR model simulations, which served as a benchmark, to restore the vital signs of the entire cohort of casualties to the healthy target range following hemorrhage required one unit of fluid for 23% of the casualties, two units for 32%, three units for 32%, and more than three units for 13%. Overall, these results suggest that the simulated hemorrhage scenarios induced diverse and substantial changes in vital signs, necessitating a range of optimal treatment options, which allowed us to fully assess the fluid-allocation recommendations predicted by the RL model.

### Assessment of the interventions predicted by the RL model

To assess the performance of the RL model in predicting optimal fluid-infusion interventions, we followed a 5-fold cross-validation procedure, where we divided the cohort of 160 casualties into five groups of 32 casualties each (4 groups for training and 1 group for testing) and averaged the results across the five folds of the testing group. Table 1 shows the confusion matrix and model accuracy for the testing group, indicating how well the interventions predicted by the RL model matched the theoretically optimal interventions for each casualty in the absence of fluid limitations. Overall, we obtained an average accuracy of 0.58, closely matching the accuracy of the training data (0.61), which suggested that the models were not overfitted to the training data and had the ability to generalize on unseen data. The testing accuracy of the four classes of casualties—those requiring one, two, three, and more than three units for vital-sign restoration—ranged from 0.37 to 0.73, with the lowest accuracy restricted to casualties requiring only one unit. The RL model tended to overtreat casualties with mild hemorrhage as having a more severe injury than they actually had and recommended two units instead of one. This error stemmed from the similarity in vital signs between the classes of casualties requiring one and two units of fluid. In contrast, the RL model tended to undertreat casualties requiring three units as belonging to the “>3 [NR]” class and incorrectly recommended zero units for them. This error stemmed from the similarity in vital signs between the classes of casualties requiring exactly three units and those requiring more than three units. (Because casualties requiring more than three units could not be fully restored in 90 min, to conserve fluids the RL agent’s optimal policy was to recommend zero units for this class.)

**Table 1:** Confusion matrix for the number of casualties for whom the reinforcement learning (RL) model allocated 0, 1, 2, and 3 units of fluid in the testing dataset of a 5-fold cross-validation analysis.

		Theoretical optimum (fluid units)				
		1	2	3	> 3 [NR]	
Units assigned by RL	1	2.6	0.6	0.0	0.2	
	2	4.8	6.6	1.4	0.0	
	3	0.0	2.6	6.6	1.0	
	0	0.0	0.4	2.4^†^	2.8^*^	
Total number of casualties		7.4	10.2	10.4	4.0	Grand total = 32
Accuracy		0.37	0.65	0.63	0.73	Average = 0.58

Entries for the number of casualties and accuracy represent averages across five cross-validation folds. NR, vital signs not restorable with 3 units or less.

*Because a maximum of three fluid units could be infused within the 90-min treatment horizon, casualties requiring more than three units could not be fully restored during that period (labeled “>3[NR]”). Consequently, to conserve fluids, the RL agent’s optimal policy was to recommend zero units for this class.

^†^The vital signs of casualties needing three fluid units were similar to those requiring more than three, which caused the RL agent to misclassify some of the three-unit cases as belonging to the “>3 [NR]” class and incorrectly recommend zero units for them.

### Comparison of fluid-allocation methods

#### Analysis 1

**Fig. 4:**
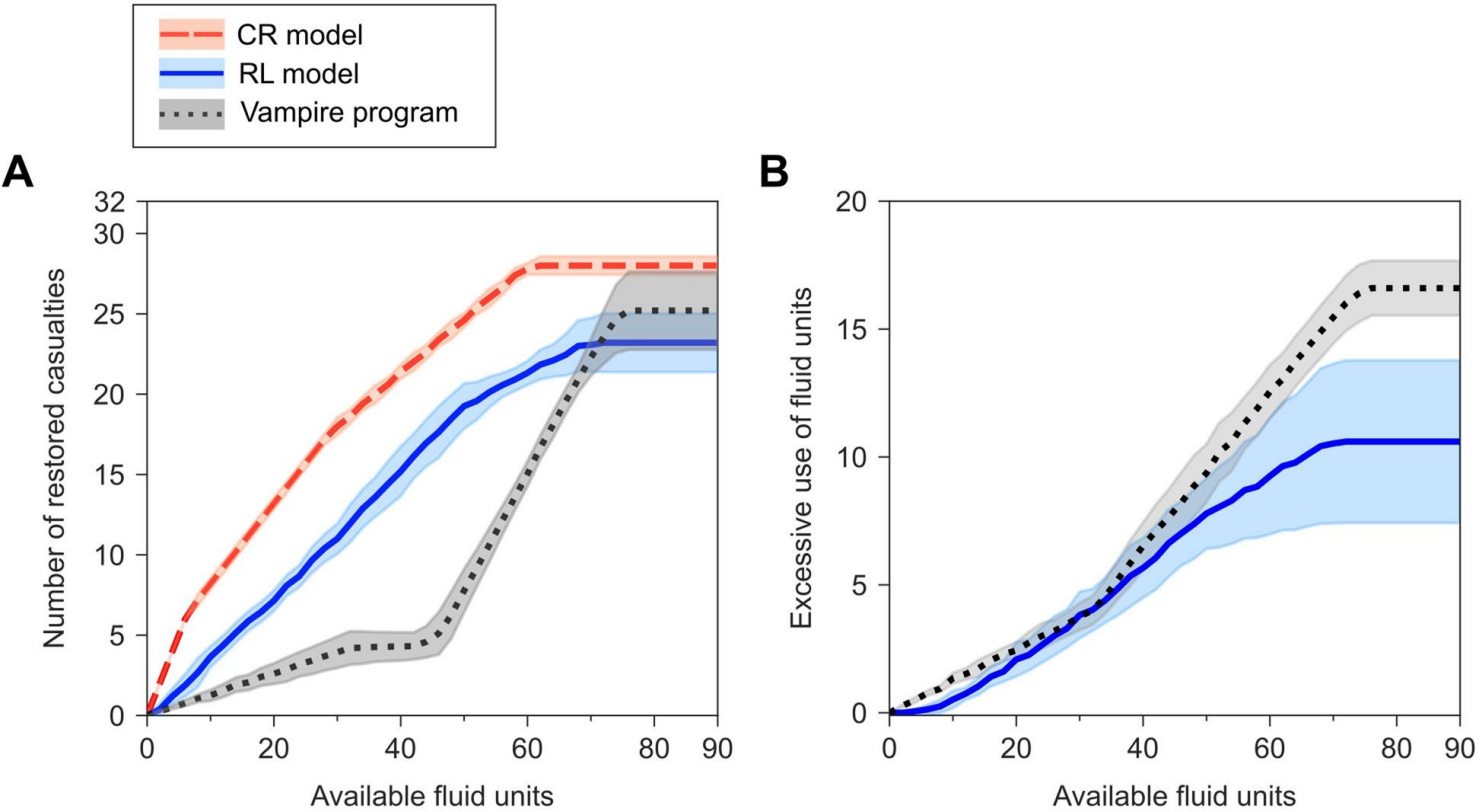
Comparison of fluid allocations for casualties (*n* = 32) in the testing dataset between the benchmark cardio-respiratory (CR) model results and the results from the reinforcement learning (RL) model and the current standard of care Vampire program. **A**) Number of casualties for whom each method restored the vital signs to the healthy target region at time *t*_*5*_. **B**) Number of fluid units used in excess of the minimum required (based on the CR model) to restore the casualties. The shaded areas represent two standard errors of the mean computed across five cross-validation folds.

Figure 4A shows that as the number of available fluid units increased, the number of restored casualties rose across all three methods. With complete knowledge of each casualty’s fluid requirement over the treatment horizon, the CR model first allocated about seven units to those requiring only one unit each, restoring approximately seven casualties at a rate of 1.0 casualties/unit. As more fluid became available, between eight and ~ 28 units, it allocated fluids to those requiring two units, restoring ~ 10 additional casualties at a rate of 0.50 casualties/unit. Between 29 and 60 units, the model allocated fluids to casualties needing three units, recovering ~ 11 additional casualties at a rate of 0.33 casualties/unit. Because the remaining four of the 32 casualties could not be restored with three units (the maximum allowed) or fewer, the total number of restored casualties plateaued at 28 once fluid availability exceeded 60 [7 + (2 × 10) + (3 × 11)] units. In contrast, the RL model restored slightly fewer casualties throughout. This was due to prediction errors (see Table 1), in particular the overestimation (and waste) of fluids required to restore casualties during the early intervention time points. The RL model restored casualties at a rate of 0.40 casualties/unit up to 50 units, after which the rate declined and plateaued at 23 restored casualties.

Overall, the Vampire program restored fewer casualties than the RL model, with the difference especially noticeable under resource-limited conditions (i.e., fewer than 60 available units for treating 32 casualties). Initially, the number of restored casualties increased at a rate of 0.13 casualties/unit until 32 units, resulting in only four restored casualties compared to 12 casualties restored by the RL model using the same amount of fluid. This poor performance is due to the Vampire program’s reliance solely on current vital signs to guide fluid allocation. At the first intervention time *t*_*2*_, none of the casualties had healthy vital signs, prompting the program to allocate one unit to each of the 32 casualties, without accounting for their future vital-sign trajectories—information the RL model used to make more suitable decisions. Because only four of the 32 casualties could actually be restored with only one unit at *t*_*2*_, the program restored only four casualties with 32 units. As more units became available, the program allocated the additional units at the second intervention time point *t*_*3*_ to 12 casualties who still had unhealthy vital signs. However, because most of these casualties required at least three units in total, receiving only two units was insufficient, causing the number of restored casualties to remain almost flat between 33 and 44 units. Beyond 44 units, the program could then allocate fluids at the third intervention time point *t*_*4*_, where 90% of the casualties again had unhealthy vital signs. Therefore, from 46 to 75 units, the number of restored casualties increased steadily at 0.67 casualties/unit, at which point the count plateaued at 25, three fewer than that of the CR model. These three casualties actually required three units each in order to be restored at *t*_*5*_, however, the Vampire program did not treat them at *t*_*3*_ because at that time they had healthy vital signs.

We also evaluated the excessive use of fluids by the two allocation methods for a fixed number of 32 casualties. Figure 4B shows the excessive use of fluids by the two methods beyond what was necessary (based on the CR model) to restore the casualties. For both methods, the excessive or inefficient use of fluid increased with fluid availability, because the inefficient use of fluids in the treated casualties accumulated with the additional treatment of each casualty. For both methods, the excessive use of fluid units increased roughly at a rate of 0.12 per available fluid units up to ~ 30 units. Between 30 and 70 available units, the rate remained the same for the RL model but it increased sharply for the Vampire program, resulting in a substantial increase in inefficient use of fluid by the Vampire program compared to that of the RL model beyond 30 units of available fluid.

### Analysis 2

Next, we examined fluid allocation while varying both the number of available fluid units (0–90) and the number of casualties (4, 8, 16, and 32) requiring fluid-infusion intervention. Table 2 shows the fraction of casualties restored to the healthy target range using the RL model relative to the Vampire program. The results consistently demonstrated that the RL model was more efficient (fraction > 1.00) in resource-limited conditions (bold font) across different numbers of casualties. In the case of 32 casualties, corresponding to the data shown in Fig. 4A, the fraction of restored casualties increased steadily with available fluids until reaching 40 units, peaking at 3.84, indicating that the RL model restored 284% more casualties than the Vampire program. This steady increase occurred because the number of casualties restored by the RL model rose steadily with available fluid, while the number restored by the Vampire program increased at a much slower rate (Fig. 4A). However, beyond 40 units, the fraction of restored casualties began to decline, and the two allocation methods became comparable when a larger number of fluid units were available, resulting in a fraction of 0.93 for 76 or more units. Averaging the ratios across the different fluid availability levels and number of casualties, we obtained a value of 2.3, indicating that the RL model restored more than twice as many casualties as the Vampire program.

**Table 2:** Fraction of casualties with vital signs restored to the healthy target range based on the reinforcement learning (RL) model relative to the Vampire program.

		Available fluid units										
		2	4	8	16	28	32	40	52	64	76	90
Number of casualties	32	**1.18** **(1.16)**	**2.08** **(0.75)**	**2.39** **(0.42)**	**3.01** **(1.07)**	**3.26** **(1.58)**	**3.30** **(1.64)**	**3.84** **(1.41)**	**2.21** **(0.43)**	1.23 (0.07)	0.93 (0.14)	*
	16	**2.64** **(0.74)**	**3.23** **(1.05)**	**3.36** **(1.55)**	**3.36** **(1.75)**	**1.67** **(0.19)**	1.20 (0.06)	0.93 (0.14)	*	*	*	*
	8	**2.99** **(0.87)**	**2.94** **(1.04)**	**3.13** **(1.46)**	1.21 (0.08)	0.93 (0.14)	*	*	*	*	*	*
	4	**2.96** **(1.09)**	**3.18** **(1.64)**	1.16 (0.09)	0.93 (0.14)	*	*	*	*	*	*	*

Fraction > 1.00 indicates that the RL model restored more casualties than the Vampire program. Resource-limited conditions are indicated in bold font determined based on the theoretical optimum, using the cardio-respiratory model. Data are presented as mean (standard deviation) of the ratios of the number of casualties restored by the RL model relative to those restored by the Vampire program for different numbers of casualties and available fluid units.

*Indicates that the values are equal to the value on their left.

Finally, to evaluate fluid-utilization efficiency, we compared the relative efficiency $\textit{R}\:$between the two methods. Table 3 shows $\textit{R}$ values for varying numbers of available fluid units and casualties requiring fluid-infusion interventions. We observed that $\textit{R}$ ranged from 1.15 to 3.85, indicating a consistently higher fluid-utilization efficiency for the RL model compared to the Vampire program. Table 3 also shows that, for each number of casualties, the relative efficiency increased steadily with the number of available fluid units before decreasing and stabilizing at 1.15. Averaging the $\textit{R}$ values across the different fluid availability levels and number of casualties, we obtained a value of 2.4, indicating that on average, per unit of fluid consumed, the RL model restored more than twice as many casualties as the Vampire program.

**Table 3:** Relative fluid-utilization efficiency $\textit{R}$ for the reinforcement learning (RL) model compared to the Vampire program.

		Available fluid units										
		2	4	8	16	28	32	40	52	64	76	90
Number of casualties	32	**1.18** **(1.16)**	**2.08** **(0.75)**	**2.39** **(0.42)**	**3.01** **(1.07)**	**3.26** **(1.58)**	**3.30** **(1.64)**	**3.85** **(1.41)**	**2.29** **(0.48)**	1.39 (0.12)	1.15 (0.07)	*
	16	**2.64** **(0.74)**	**3.23** **(1.05)**	**3.36** **(1.55)**	**3.38** **(1.74)**	**1.81** **(0.26)**	1.39 (0.11)	1.15 (0.07)	*	*	*	*
	8	**2.99** **(0.87)**	**3.03** **(1.08)**	**3.29** **(1.57)**	1.40 (0.10)	1.15 (0.07)	*	*	*	*	*	*
	4	**3.14** **(1.10)**	**3.42** **(1.84)**	1.38 (0.09)	1.15 (0.07)	*	*	*	*	*	*	*

$\textit{R}$ >1.00 indicates that the RL model restored more casualties per unit of fluid consumed than the Vampire program. Resource-limited conditions are indicated in bold font determined based on the theoretical optimum, using the cardio-respiratory model. Data are presented as mean (standard deviation) of $\textit{R}$ for the different numbers of casualties and available fluid units.

*Indicates that the values are equal to the value on their left.

The sensitivity analysis indicated that small errors in vital-sign measurements had minimal, if any, impact on fluid-allocation decisions. For example, over the entire range of available fluid units in Fig. 4A, the root-mean-square error (RMSE) between the number of casualties restored by the RL model with measurement noise and without (Fig. 4A, solid line) was 0.6 casualties. Similarly, for the Vampire program, the RMSE value was 0.9 casualties. Despite the introduced noise, the RL model still restored 2.4 times more casualties overall and 2.5 times more casualties per unit of consumed fluid than the Vampire program. These results strongly suggest that the proposed fluid-allocation method is robust to small errors in vital-sign measurement.

## Discussion

Future armed conflicts are expected to involve large-scale operations with mass casualties^27^, where combat medics may need to prioritize care and fluid resuscitation. However, the U.S. Department of Defense guidelines for fluid resuscitation have not been designed to address resource-constrained conditions^6^. Here, we leveraged AI methods to provide automated medical decision support that optimizes resource utilization by treating the largest number of casualties under constrained fluid-resuscitation resources. We developed and evaluated these methods using synthetic vital-sign data that had sufficient fidelity to represent hemorrhagic injury and treatment scenarios typically encountered in battlefield trauma. Our simulation results suggest that, on average, under varying levels of resource constraints, the investigated AI fluid-allocation method restored more than twice as many casualties as the allocation based on current U.S. military standard of care guidelines.

A crucial yet challenging step in optimal resource allocation is projecting resource requirements over the entire treatment horizon. In our previous work^8^, we addressed this by using an AI RNN to predict vital-sign trajectories for all possible treatment options, from which we selected the optimal one. This is a viable approach when the treatment horizon is relatively short and the number of possible treatment options is limited. However, when the algorithm has to assess a large number of potential types of fluid^28^ over a long treatment horizon, the number of possible treatment combinations increases exponentially, making the RNN approach increasingly complex. To develop a more scalable method and simplify the number of projections, we combined an RL model with a linear regression model, which enabled us to directly compute a single sequence of optimal interventions for the entire treatment horizon, irrespective of the number of fluid types involved. In addition, unlike RNNs, which require a time series of vital signs to determine optimal interventions, the RL model needs only a single vital-sign measurement at the intervention time. This makes the RL model more robust to field conditions, where complete time-series data may not be available.

Using synthetic trauma casualties and simulated battlefield trauma scenarios, we developed and assessed the ability of the RL model to optimally allocate limited fluid resources and compared its performance with the current standard of care, the Vampire program. The use of synthetic data allowed us to perform a head-to-head comparison of the two methods using the same set of synthetic trauma casualties, which is impractical using real-world data. These comparisons showed that across every comparison the RL model outperformed the current standard of care. Importantly, the RL model showed consistent improvement over the Vampire program under resource-limited conditions. The main reason for this improvement is that, while the Vampire program only considers vital signs at the current intervention time, the RL model prognosticates their future values to make informed decisions at the current time while considering the entire treatment horizon.

Future large-scale combat operations will likely involve mass casualties, delayed evacuations, and prolonged casualty care, placing considerable strain on medical resources. Our AI framework can be readily adapted to some of these scenarios by considering additional resuscitation-fluid types and long treatment horizons. For example, using the latest version of the CR model^28^, we could simulate vital-sign responses to six fluid types, including freeze-dried plasma, which may serve as an initial bridge for future whole blood transfusion^29,30^. The increase in the number of fluid types would linearly increase the number of options investigated by the RL model, where at each decision point it would select an optimal action from among *K* + 1 options, where *K* represents the number of available fluid types. To accommodate long treatment horizons, the RL model could include additional decision time points, while the representation of the state of the casualty could be enhanced by incorporating additional physiological indicators, such as tissue oxygen debt represented by lactate and base deficit levels, to capture evolving pathophysiology^31,32^.

### Limitations

Our study has limitations stemming from both methodological and practical constraints. First, we relied on synthetic data rather than real-world trauma casualty data to develop the RL model. As a result, the data cannot fully capture all the complexities of actual trauma scenarios. However, the CR model has been validated to adequately capture the dynamics of hemorrhage and related treatments, enabling the development of the RL model to solve complex fluid-allocation problems. Given the scarcity of clinical data from trauma casualties and the inherent difficulties in collecting such data, leveraging synthetic data remains a necessary and practical approach for developing automated decision-support systems^33,34^. Second, casualty mortality instead of vital-sign stabilization should be the desired end point for prioritizing fluid-resuscitation treatment, because casualties requiring fewer units of fluid are more likely to survive without treatment than casualties requiring a larger number of units. However, predicting casualty mortality is challenging because it depends on numerous factors, which cannot be readily accounted for. Therefore, using vital-sign thresholds beyond which fluid transfusion is not needed based on the current U.S. military standard of care was a reasonable compromise. Third, our assumption that the bleeding rate will become zero upon tourniquet application, with no further uncontrolled bleeding or medical interventions, may not be realistic. However, this assumption enabled us to simplify and solve the complex fluid-allocation problem. Fourth, while the proposed approach can be expanded to consider multiple rebleeding episodes, this would require advance knowledge of the potential rebleeding scenarios to appropriately train the model. Finally, the version of the CR model we used does not incorporate the effects of different types of fluids. Consequently, the RL model was trained using a generic fluid volume. This limitation can be rectified in future studies by using the latest version of the CR model, which can simulate vital-sign trajectories for infusion of six different fluid types^28^, including those recommended by the TCCC guidelines^7^.

## Conclusions

AI tools are increasingly being adopted to solve diverse medical problems^35–37^. However, these powerful technologies often face challenges, such as data bias and poor generalizability due to overfitting. Here, using synthetic data that represented a diverse set of bleeding and treatment scenarios after a traumatic injury, we developed an AI method to optimize fluid allocation under mass-casualty conditions and showed that the method had the ability to generalize on unseen data. Although this approach does not fully replicate real-world complications of hemorrhage, the study design allowed us to develop insights into the challenges and opportunities for improving medical decision-making in austere conditions using AI technologies. Based on simulations, our methodology demonstrated an efficient use of limited fluids, with more than twice as many casualties restored compared to the current standard of care.

As revealed by the ongoing conflict in Ukraine^38^, treatment and evacuation capacities are likely to be severely crippled in future conflicts. These conditions necessitate that pre-hospital treatments be extended for hours, if not days^39,40^, requiring medics to continuously triage combat casualties and decide between different types of fluids for treatment. Therefore, it would be highly beneficial to extend and evaluate RL models for allocating multiple fluid types and medical interventions over long treatment horizons. Even more than medical supplies, caregivers—medics, nurses, and medical doctors—are often the most constrained resource in mass-casualty incidents in civilian and military settings^1^. Our study presents a promising technology for alleviating the excessive cognitive burden on caregivers under these circumstances by providing the means to triage casualties in a resource-constrained environment.

## Supplementary Information

Below is the link to the electronic supplementary material.

Supplementary Material 1

## Data Availability

Data used in this study will be made available following a written request to the corresponding author, along with a summary of the planned research.
