# Supplementary material for: A reinforcement learning algorithm to optimize resource utilization in combat casualty care
Source: Sci Rep. 2025 Dec 24;15:44534. doi: 10.1038/s41598-025-28021-6 (PMC12738704; doi:10.1038/s41598-025-28021-6)
Supplement: Supplementary file 1 — Supplementary Material 1 [file 41598_2025_28021_MOESM1_ESM.docx]

**A reinforcement learning algorithm to optimize resource utilization in combat casualty care**

Manivannan Subramaniyan^1,2^, Xin Jin^1,2^, Sridevi Nagaraja^1,2^, Anders Wallqvist^1^, and
Jaques Reifman^1*^

^1^Department of Defense Biotechnology High Performance Computing Software Applications Institute, Defense Health Agency Research & Development, Medical Research and Development Command, Fort Detrick, MD, USA

^2^The Henry M. Jackson Foundation for the Advancement of Military Medicine, Inc., Bethesda, MD, USA

*Address correspondence to:

Jaques Reifman, Ph.D., Senior Research Scientist and Director

Department of Defense Biotechnology High Performance Computing Software Applications Institute

Defense Health Agency Research & Development

Medical Research and Development Command

ATTN: FCMR-TT, 504 Scott Street

Fort Detrick, MD 21702-5012

Tel: (301) 619-7915; Fax: (301) 619-1983

Email: jaques.reifman.civ@health.mil

Supplementary Information

Selection of timelines for the hemorrhage and treatment scenarios

When creating the hemorrhage and treatment scenarios, we used a combination of fixed and variable time durations for the different events to capture the temporal variability for treatment of combat casualties in a pre-hospital setting [1]. Specifically, we followed the fixed 5 min of uncontrolled bleeding with tourniquet application after a variable delay of 0–10 min, consistent with the average tourniquet application time in recent conflicts [2]. The variable 10- to 15-min period following tourniquet application accounted for the preparation for fluid infusion starting at *t_2_* (Figure 2). Together, these time intervals enabled us to adhere to military guidelines, which recommend the initiation of fluid infusion within 30 min of a traumatic injury [3].

Parameter-set generation to simulate unique synthetic casualties

We represented unique trauma casualties in the cardio-respiratory (CR) model by varying the model parameters and representing different levels of hemorrhage. To this end, we generated unique parameter sets for the CR model by randomly sampling the 74 parameter values within ±70% of their nominal values. Then, we down selected the sets by eliminating those that produced *1*) vital signs that were not in the physiological range [40 < heart rate (HR) < 200 beats/min and 40 < systolic blood pressure (SBP) < 260 mmHg] according to vital-sign monitors [4, 5], *2*) oscillations in vital signs, and *3*) initial vital signs at *t_2_* in the healthy range based on the Vampire program (HR ≤ 100 beats/min and SBP ≥ 100 mmHg) [1], because these casualties would not need fluid-infusion intervention. We also removed certain parameter sets to ensure that the resulting vital-sign trajectories were sufficiently distinct from each other.

Reinforcement learning model

Using a reinforcement learning (RL) model, we aimed to identify a sequence of fluid-infusion interventions at times *t_2_*, *t_3_*, and *t_4_* that restores each casualty’s vital signs to the healthy target region by the end of the treatment scenario at time *t_5_*, while using the least amount of fluid possible. To achieve this goal, we used Q-learning to train Q-networks as described previously [6, 7], with state $\text{s}$, action $\text{a}$, environment, and reward$\text{r}$ defined as follows.

*Action*

We defined two discrete actions $\text{a}\text{ }\text{=}\text{ }\text{0}$ and $\text{a}\text{ }\text{=}\text{ }\text{1}$, where 0 indicates no infusion of fluids for the next 30 min and 1 indicates infusion of 1 unit of fluid over the next 30 min.

*State*

The state $\text{s}_{\text{t}}$ at an intervention time point *t* $=$ *t_m_* is an 11-element vector consisting of HR and SBP values at times *t_m_* and *t_m-1_*, with the past two actions represented in one-hot encoded form [e.g., the sequence of the past two actions 0 and 1 is coded as (0, 1, 0, 0)] and the index of the intervention time point also one-hot encoded [e.g., for an intervention at time *t_3_*, the time-index value 3 is encoded as (0, 0, 1)]. Because a single measurement of HR and SBP at a given time point does not have sufficient information for an agent to take an optimal action, we defined the state to include the past vital signs and actions, as previously reported [7]. For each casualty, we defined three states—*s_t2_*, *s_t3_*, and *s_t4_*—corresponding to times *t_2_*, *t_3_*, and *t_4_*, respectively, at which the agent took actions, and a terminal state *s_t5_* corresponding to time *t_5_*, at which the agent did not take any action. Because at the initial intervention time $\text{t}_{\text{2}}$ there was no historical information available to construct state *s_t2_*, we set the past two actions to zero, as no fluid had been infused before *t_2_*, and we used HR and SBP values at 10 min before *t_2_* as the vital signs at the “previous” intervention time point. Similarly, for state *s_t3_*, one of the two past actions did not exist, so we set it to zero.

*Environment*

The CR model served as the *environment* in the RL formalism. Specifically, in response to the RL agent’s actions, we applied the corresponding fluid infusion within the CR model to generate synthetic vital-sign data for the next state. For example, if the agent took action 1 for a casualty at state *s_t2_*, the CR model simulated a 30-min fluid infusion starting from *t_2_*, and the vital signs at the end of the 30-min infusion were used to construct the next state *s_t3_*. This process illustrates how we achieved state transitions.

*Reward*

We computed the reward $\text{r}$ using a function that favored actions that improved the resulting vital signs and discouraged actions that increased fluid usage. Accordingly, we defined the reward function as follows:

| $\text{r}\text{(}$*s_t_*, $\text{a}$, *s_t+1_*$\text{)}=\text{∆d}-\text{w}_{\text{1}}\text{a}+\text{w}_{\text{2}}\text{H}$ | (1) |
| --- | --- |
| $\text{∆d}= \text{d}\text{(}$*s_t_*$\text{)}- \text{d}\text{(}$*s_t+1_*$\text{)}$ | (2) |
| $H= \left\{ \begin{aligned} \text{+1, if }\text{s}_{\text{t+1}}\text{= }\text{s}_{\text{t5}}\text{ and vital signs were restored } \\ \text{-1, if }\text{s}_{\text{t+1}}\text{=}\text{ s}_{\text{t5}}\text{ and vital signs were not restored } \\ \text{0, otherwise}\text{ }\text{ } \end{aligned} \right.$ |  |

where the scalar value $\text{∆d}$ denotes the improvement in the vital signs, with $\text{d}\text{(}$*s_t_*$\text{)}$ representing the Euclidean distance between the current vital signs at time $\text{t}$ and the nearest point in the healthy target region for vital signs (HR ≤ 100 beats/min and SBP ≥ 100 mmHg). We computed $\text{d}\text{(}$*s_t+1_*$\text{)}$in a similar manner for the next state *s_t+1_*$.$ The scalar parameters $\text{w}_{\text{1}}$ and $\text{w}_{\text{2}}$ weighted fluid usage and the casualty-treatment outcome $\text{H}$, respectively.

To begin training the Q-network, for each casualty in the training dataset, the agent started at state *s_t2_*, took an action $\text{a}$, received reward $\text{r}$, and ended up in the next state *s_t3_*. The agent then repeated this process until reaching the terminal state *s_t5_*. This set of transitions starting from the initial state *s_t2_* to the terminal state *s_t5_* defined an *episode*, and each tuple (*s_t_*, $\text{a}\text{,} \text{r}\text{,}$*s_t+1_*) constituted an “*experience*” of the agent. We used these experiences to train a feedforward artificial neural network (ANN) by minimizing an objective function of the form $\left[ \text{Q}_{\text{target}}-\text{Q}\text{(}\text{s}_{\text{t}}\text{,}\text{ }\text{a}\text{)} \right]^{\text{2}}$, where $\text{Q}\text{(}\text{s}_{\text{t}}\text{,}\text{ }\text{a}\text{)}$ denotes the output of the ANN and $\text{Q}_{\text{target}}=\text{r}+\text{γ}\max_{a^{'}} \text{Q}\left( \text{s}_{\text{t+1}}\text{, }\text{a'} \right)$ denotes the target value, with $\text{γ}$ representing a discount factor. Specifically, we used the double deep Q-network (DDQN) architecture as our ANN, in which, in addition to the Q-network used to estimate $\text{Q}^{*}\text{(}\text{s}\text{,}\text{ }\text{a}\text{)}$, we employed additional Q-networks [6]. Each Q-network consisted of two hidden layers with 64 and 32 units, had leaky-ReLU activations, and had batch normalization applied to the outputs of each layer. Using the Keras framework in Python [8], we trained the DDQN using experience replay [7] and a batch size of 32. Each time the agent took an action, we updated the Q-network parameters using 256 randomly sampled agent experiences accumulated up until that time point. We terminated the training process after a predetermined number of learning episodes, which was a hyperparameter.

We performed hyperparameter tuning to determine optimal values for the number of learning episodes and the weight parameters $\text{w}_{\text{1}}$ and $\text{w}_{\text{2}}$ in the reward function. Specifically, for different combinations of the hyperparameters, we used 75% of the training dataset to train the RL models and validated their performance on the remaining 25% of the training data not used for model training. We then selected the combination of hyperparameters that maximized model performance. We conducted the hyperparameter search over the range of 0.4–1.1 for $\text{w}_{\text{1}}$ and $\text{w}_{\text{2}}$ and 128–320 for the number of learning episodes.

**RL model assessment**

After training the RL model, we assessed its ability to predict the optimal actions at times *t_2_*, *t_3_*, and *t_4_* to restore each casualty at time *t_5_* using the least amount of fluids. To this end, for each of the casualties in the testing dataset, we computed the sequence of optimal actions using the following two steps: *1*) starting at time *t_2_*, we computed $\text{Q}^{*}($*s_t_*,$\text{a})$ values for actions $\text{a}=0$ and $\text{a}=\text{1}$by providing the state *s_t2_* as input to the Q-network and then selected the action (0 or 1) that resulted in the higher output value. Next, we applied the selected fluid-intervention action as input to the CR model and obtained the vital-sign values corresponding to time *t_3_*. *2*) At times *t_3_* and *t_4_*, we sequentially applied the process in the step above for states *s_t3_* and *s_t4_* and obtained the optimal actions corresponding to times *t_3_* and *t_4_*, respectively. To assess these model predictions, we computed the fraction of casualties for whom the predicted sequence of actions matched the theoretical optimum. Note that multiple action sequences [e.g., (0, 1, 1) and (1, 1, 0), each corresponding to decision times *t_2_*, *t_3_*, and *t_4_*, respectively] could be optimal for a given casualty. We counted the RL model-predicted action sequence as correct if it matched any one of the theoretically optimal sequences of actions.

References

1. Voller, J. et al. Joint Trauma System Clinical Practice Guideline (JTS CPG): prehospital blood transfusion. 30 October 2020. *J. Spec. Oper. Med.* **21**, 11–21, doi:10.55460/p685-l7r7 (2021).

2. Kragh, J. F., Jr. et al. Survival with emergency tourniquet use to stop bleeding in major limb trauma. *Ann. Surg.* **249**, 1–7, doi:10.1097/SLA.0b013e31818842ba (2009).

3. Shackelford, S. A. et al. Joint Trauma System, Defense Committee on Trauma, and Armed Services Blood Program consensus statement on whole blood. *Transfusion* **61**, Suppl 1, S333–S335, doi:10.1111/trf.16454 (2021).

4. Mazoteras-Pardo, V., Gómez-Cantarino, S., Ramírez-Jiménez, M., Navarro-Flores, E. & Ugarte-Gurrutxaga, M. I. Validations of blood pressure measuring devices using recognized protocols. *J. Pers. Med.* **13**, 9, doi:10.3390/jpm13010009 (2022).

5. Peprah, Y. A., Lee, J. Y. & Persell, S. D. Validation testing of five home blood pressure monitoring devices for the upper arm according to the ISO 81060-2:2018/AMD 1:2020 protocol. *J. Hum. Hypertens.* **37**, 134–140, doi:10.1038/s41371-022-00795-6 (2023).

6. Hasselt, H. V., Guez, A. & Silver, D. Deep reinforcement learning with double Q-Learning. *Proceedings of the 30th Association for the Advancement of Artificial Intelligence Conference, 2016, Phoenix, AZ*; 2094-2100.

7. Mnih, V. et al. Human-level control through deep reinforcement learning. *Nature* **518**, 529–533, doi:10.1038/nature14236 (2015).

8. Chollet, F. Keras. (2015). Available at https://github.com/fchollet/keras, accessed on October 30, 2025.
